# Characteristics and impact of aged coal ash with slag emplaced in a karst cave: the case of Divaška jama, Slovenia

**DOI:** 10.1038/s41598-021-02842-7

**Published:** 2021-12-03

**Authors:** Andreea Oarga-Mulec, Sara Skok, Tatjana Simčič, Janez Mulec

**Affiliations:** 1 University of Nova Gorica, Vipavska cesta 13, 5000 Nova Gorica, Slovenia; 2 Research Centre of the Slovenian Academy of Sciences and Arts, Karst Research Institute, Titov trg 2, 6230 Postojna, Slovenia; 3 National Institute of Biology, Večna pot 111, 1000 Ljubljana, Slovenia; 4 UNESCO Chair on Karst Education, University of Nova Gorica, Glavni trg 8, 5271 Vipava, Slovenia

**Keywords:** Environmental sciences, Natural hazards

## Abstract

A mixture of coal bottom ash and slag, with a fraction of fly ash (CAFAS) from steam locomotives, was placed in the cave Divaška jama to delimit and level tourist trails. Emplacement began in 1914 and carried on for several decades. The CAFAS mixed with other cave material gradually changed its structure and appearance. Currently the concentration of some elements in the CAFAS (As, Cu, Hg, Ni, Pb, Zn), and also to a lesser extent in cave sediments (Cr, Cu, Ni), indicates a possibly harmful effect on sediment-associated biota based on ecotoxicological assays. Compared to the cave sediment, the CAFAS contains distinctly different mineral phases and presents a different source of radioactivity. Microbial metabolic activity of CAFAS is low, 0.22 μl O_2_/gDW h, but higher than that of cave sediment. The present environmental hazards from CAFAS are estimated to be low. Whereas the emplacement of CAFAS was seen initially a long-term solution for waste disposal and management of the cave, it turned out that CAFAS enriches the underground environment with inorganic and organic compounds and disperses pollution into the cave ecosystem. After its removal from the cave, the CAFAS should be investigated thoroughly due to its susceptibility to alteration.

## Introduction

Combustion of coal produces gas emissions and solid by-products in the form of fly ash, bottom ash and boiler slag. Coal ash (CA), especially bottom ash from steam power generation, was one of the first massively produced wastes related to the Industrial Revolution. CA contains Al_2_O_3_, CaO, Fe_2_O_3_, K_2_O, MgO, Na_2_O and SiO_2_ as its major mineral components^1^; others are present in small quantities, depending largely upon the properties of each individual coal^2^. Polycyclic aromatic hydrocarbons (PAHs) from unburned or incompletely burned organic matter remain within the coal ash^3^. Most of the CAs, once considered harmless and hence deposited widely in landscapes, landfills and mines, are now the subject of controversial discussions about the hazards they might present^4^. Residual coal dust and combustion by-products within the ashes have a genotoxic and mutagenic effect on biota, related to the release of PAHs and heavy metals^5^. When stored in settling ponds in the immediate vicinity of waterways, CA poses a considerable risk to both surface water and groundwater^6^. It is estimated that more than 9000 ash ponds are in operation worldwide^7^. Technical failure of such ponds can have catastrophic consequences, as in the case of the Kingston power plant in Tennessee, USA, in 2008^8^ or in the case of Sutton Lake, USA, where uncontrolled CA spills occurred from impoundments and landfills in flood-prone areas^9^. In a study eight years after the spill, CA was found to be buried beneath natural sediment (13–18 cm) in a nearby creek^10^. High concentrations of ash, metals, and metalloids were still present in the shallow sediments (0–3 cm) near the Kingston site, indicating redistribution of CA from upstream. It was also found that metals and metalloids associated with CA were not mobilized by river water. Previous studies have shown that certain conditions in water and river sediments can control the mobilization of certain pollutants from coal combustion residues. For example, As and Se are mobilized under reducing and oxidizing conditions, respectively. Low pH affects the leachability of metals and high pH of metalloids; ORP and pH together determine the speciation and mobility of elements from coal combustion residues^11^. It is well known that contamination of water supplies by CA poses a physical threat to animal and human life^12,13^. Research and case studies have highlighted the harmful long-term effects of bioaccumulation of metalloids in the ecosystem^14^, particularly selenium, which bioaccumulates in aquatic animals and plants at the base of the food chain^15^.

Historically the increasing production of CAs soon became a problem, not only near industrial centres but also along the railway lines where they were dropped from steam locomotives. Most of the ejected material consisted of coal bottom ash and slag and a part of fly ash. To remove part of this waste from the landscape, in 1914 some material was introduced into a karst cave at Divača, Slovenia, where it was used to help develop tourist trails^16^. It was deposited, mostly along the trails, on top of slippery natural clay. During the decades that followed, about 100 m^3^ of this waste was brought into the cave. By mixing with cave sediments, debris and infiltrates at high humidity, coal bottom ash and slag with a fraction of fly ash gradually turned into blackish dirt (hereafter CAFAS). Hardly any data relating to the long-term effects of CAFAS on groundwater, karst caves, karst ecosystems and accompanying biota are available. Some studies of negative impacts upon local ecosystems, ranging from the disruption of natural drainage systems to the absence of vegetation due to nutrient deficiency, poor soil structure, acidity and metal toxicity, have been conducted at abandoned mines in various countries^17–19^.

The current study addressed the ongoing leaching behaviour of CAFAS, unique material, from Divaška jama, and its toxicity and microbial activity compared to natural cave sediment. The results are important to aid estimation of the long-term effects of allochthonous CAFAS waste on groundwater and the underground ecosystem when deposited under stable environmental conditions of high humidity and low temperature, and also to advise planning of management alternatives.

## Results and discussion

### Chemical characteristics and environmental impact

**Figure 1:**
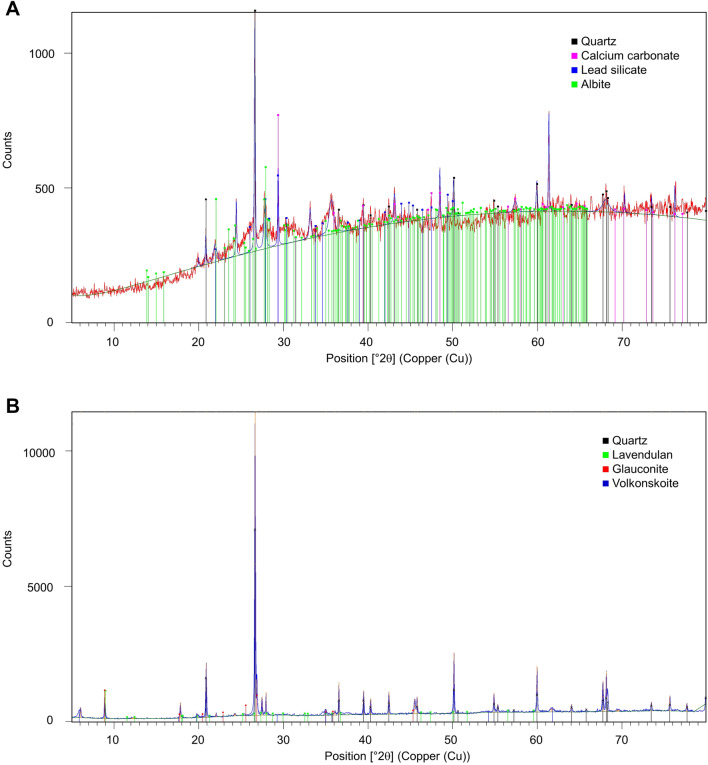
Diffractograms of CAFAS (**A**) and cave sediment (**B**).

**Figure 2:**
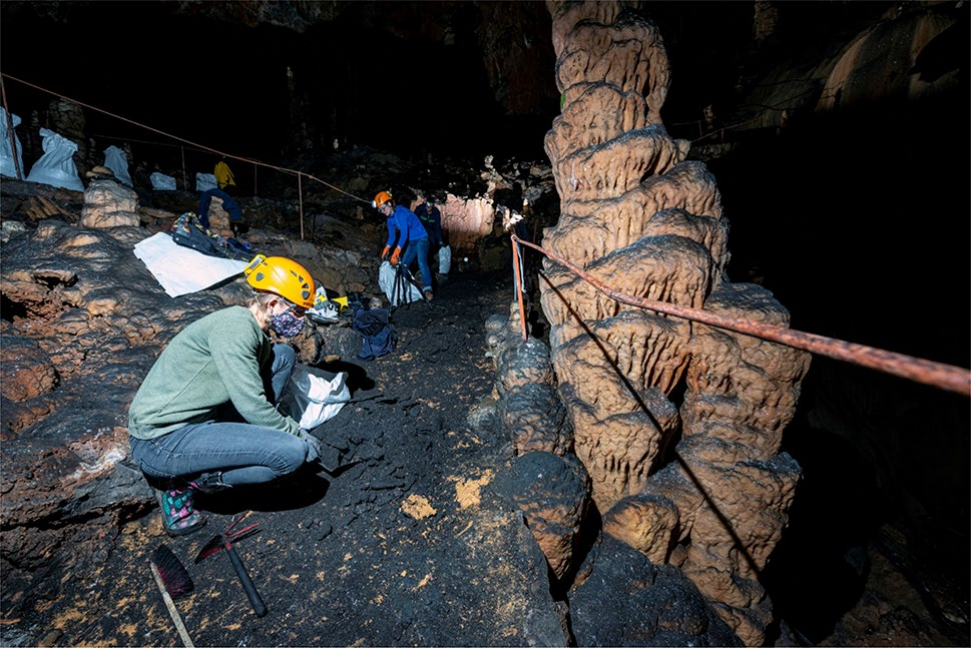
Removal of deposited CAFAS on tourist footpath in Divaška jama, 2019 (Photo: courtesy by Borut Lozej).

CAFAS introduced into Divaška jama has changed the appearance and natural conditions of the cave (Fig. 2). To estimate its current impact on the cave, CAFAS was compared with sediment derived from eroded bedrock, deposited in the cave during the Pleistocene. Although the two samples did not have the same origin, some elements in both showed similar concentrations, e.g. Al, Be, Cd, Fe, Mg, V; some were comparatively higher within the CAFAS (Ca, Cu, Na, Pb, As, Hg, Zn), and some were higher within the sediment (Ni, Mn, K, Co, Cr) (Table 1). Identified crystalline phases between natural cave sediment and CAFAS were distinctively different (Table 2). In comparison to cave sediment, the CAFAS contained a large proportion of non-crystalline amorphous phase material (Fig. 1). Chemical and biological changes occurred in the CAFAS after its introduction into the cave; in particular it became enriched in organic matter. The exact physico-chemical changes of CAFAS cannot be determined because the original material introduced into the cave is not available for analysis. In general, coal ash does not contain organic material, and if it does, it is usually unburned or incompletely burned organic material, which usually constitutes a very small fraction^20^. CA and bottom ash are formed in a combustion chamber at high temperatures and under dry and oxygen-rich conditions. When the material is exposed to the external environment, chemical changes occur as it reacts with water, which is in vapour or liquid form. This results in new mineral phases and leachate, which usually contains soluble salts, mobilised metals, and other toxic pollutants^15^. The CAFAS analysed in this study contained a significant amount of elements (C, N, P) that are critical for microbial metabolism^21^ and can be attributed to increased microbial activity in CAFAS compared to cave sediment (Table 4).

**Table 1:** Physico-chemical and radioactivity analyses of cave sediment and CAFAS from Divaška jama.

Parameter	Unit in DW	Meas. uncertainty	Method/reference	TEC for freshwater sediment*	Limit value for soil treatment**	Sediment	CAFAS
Ash	%	± 5%	ISO 11465			96.0	71.2
Dry mass	%	± 5%	ISO 11465			77.3	69.2
POM	%	± 5%	Dean 1974^22^			3.2	37.0
pH		± 0.1 pH	ISO 10390			7.20	7.29
C	%	± 15%	ISO 10694			3.96	28.8
N	%	± 15%	ISO 5663			0.28	0.69
P	g/kg	± 20%	ISO 11263			0.46	0.76
S	%	± 20%	ISO 15178			0.007	< 0.007
Al	mg/kg	± 20%	ISO 12020			42,100	38,600
As	mg/kg	± 20%	ISO 15586	9.79		8.57	**15.0**
Be	mg/kg	± 20%	ISO 15586			1.02	1.61
Ca	g/kg	± 20%	ČSN 46 7092			5.21	66.7
Cd	mg/kg		ISO 5961	0.99	1	< 0.41	< 0.41
Co	mg/kg	± 20%	ISO 8288			25.3	10.9
Cr	mg/kg	± 20%	ISO 5961	43.4	100	**109**	39.5
Cu	mg/kg	± 20%	ISO 5961	31.6	60	**48.7**	**477**
Fe	mg/kg	± 20%	ČSN 75 7385^a^			43,250	48,720
Hg	mg/kg	± 15%	ČSN 75 7440*	0.18	0.8	0.082	**0.205**
K	g/kg	± 20%	ČSN 46 7092			9.95	3.73
Mg	g/kg	± 20%	ČSN 46 7092			7.91	5.09
Mn	mg/kg	± 20%	ČSN 757385^a^			1340	474
Na	g/kg	± 20%	ČSN 46 7092			0.19	2.43
Ni	mg/kg	± 20%	ISO 5961	22.7	50	**130**	**32.4**
Pb	mg/kg	± 20%	ISO 5961	35.8	85	11.8	**162**
V	mg/kg	± 20%	ISO 15586			78.3	95.8
Zn	mg/kg	± 20%	ISO 5961	121	200	97.9	**205**
Radiation	cpm					65	63
**Radionuclide**							
^238^U (1)	Bq/kg					33.0 ± 6.8	153.7 ± 26.8
^226^Ra (1)	Bq/kg					23.9 ± 1.0	119.6 ± 2.0
^210^Pb (1)	Bq/kg					19.7 ± 3.4	140.0 ± 11.5
^228^Ra (2)	Bq/kg					35.0 ± 1.3	33.3 ± 1.2
^212^Pb (2)	Bq/kg					33.7 ± 1.2	33.9 ± 1.2
^228^Th (2)	Bq/kg					30.7 ± 1.5	27.9 ± 1.3
^40^K	Bq/kg					476.5 ± 26.7	115.8 ± 8.0

The stated measurement uncertainty is the product of the standard measurement uncertainty and the expansion coefficient k = 2, which corresponds approximately to 95% coverage probability for normal distribution. Values shown in bold are those that exceed either threshold effect concentration (TEC) below which harmful effects are unlikely to be observed in freshwater ecosystems (MacDonald et al.^23^) (*) and/or limit values for heavy metals in soil set by Decree on the management of sewage sludge from urban waste water treatment plants, Uradni list RS 2008, 62/08 (**).

*ČSN* National standards of the Czech Republic; ^a^Flame atomic absorption spectrometric method; *Determination of total mercury by thermal decomposition, amalgamation and atomic absorption spectrometry; (1) uranium decay chain, (2) thorium decay chain.

**Table 2:** Identified crystalline phases of cave samples.

Sediment	CAFAS
Quartz (SiO_2_)	Quartz (SiO_2_)
Lavendulan (NaCaCu_5_(AsO_4_)_4_Cl·5H_2_O)	Calcium carbonate (CaCO_3_)
Glauconite (K, Na)(Fe, Al, Mg)_2_(Si, Al)_4_O_10_(OH)_2_	Lead silicate (Pb_2_SiO_4_)
Volkonskoite (Ca_0.3_(Cr, Mg)_2_(Si, Al)_4_O_10_(OH)_2_·4H_2_O)	Albite (NaAlSi_3_O_8_)

The total element concentration is insufficient to estimate the environmental burden in the sediments, but the addition of data on the bioavailability, toxicity and mobility of the elements provides a more comprehensive overview^24,25^. First, the results of chemical analyses were compared with a consensus-based sediment quality guideline that defines a threshold effect concentration (TEC), below which adverse effects on freshwater sediment-dwelling organisms are unlikely^23^. Because some of the values exceeded TEC and because CAFAS is to be extracted completely from the cave and further managed, these values were compared with the current legislation to examine options for waste management. Neither CAFAS nor-even cave sediment would be a suitable soil treatment in agriculture (Table 1). Due to the origin of CAFAS, i.e. coal ash and slag, it can be classified as non-hazardous waste that should be tested for leachate behaviour and then landfilled according to legislation (Decree on waste landfill, Uradni list RS, 2014, 10/14). Considering growing environmental concerns and the limited space available for landfilling, a more attractive alternative to the disposal of such no-value waste might be recycling, with onward use in various geotechnical applications, for example as geotechnical composites^26^. Elevated concentrations of natural radionuclides are, however, commonly found in by-products of coal combustion^27^. In this study, both cave sediment and CAFAS expressed comparable low levels of radiation, but they differed in the radioactivity of natural radionuclides (Table 1). Even if CAFAS fits the legislation criteria on specific gamma irradiation for application in construction materials (Decree on limit doses, reference levels and radioactive contamination, Uradni list RS, 2018, 18/18; Decree on radiation activities, Uradni list RS, 2018, 19/18), it should still be investigated thoroughly because of its long exposure under cave conditions.

The concentrations of elements in the material provide crucial information for considerations of proper waste management. As mentioned above, the mobility and leaching of major and trace elements are additional important characteristics to help assess the potential impact on the environment and biota correctly. To simulate the effects of leaching and environmental changes with respect to the altered chemistry of infiltrating water, two different pH values were used. Greater quantities of ionic compounds were leached from CAFAS as indicated by higher electrical conductivity values. In both cases the eluates were equilibrated at the end of the experiment at a pH around neutral (Table 3). The pH influences the leaching behaviour of the toxic compounds in CA^28^. The highest leaching rate was at pH 5.21, when 0.205% Cu was mobilized from the cave sediment and 0.154% Ni from the CAFAS (Table 3). Due to the low mobility of the compounds investigated, and because the concentrations of the readily soluble fractions were generally below 0.5%, the potential environmental hazard and threat for biota and groundwater is estimated to be low. This is in line with previous findings, which show that the pollution potential of aged ash is significantly reduced compared to that of fresh and recently deposited ash^29^. It is also not expected that the current level of leaching of residual compounds from CAFAS (Table 3) will have additional negative impacts on the cave ecosystem. On the other hand, it has contributed to an unaesthetic and unnatural appearance of the cave (Fig. 2).

**Table 3:** Characteristics of water leachate and eluates with the percentage of compounds leached from cave sediment and CAFAS.

	Leachate		Eluate			
	pH	EC (µS/cm)	pH	EC (µS/cm)	K (mg/l) (%)	Cl (mg/l) (%)
Sediment	5.21	5.7	7.07	47.7	1.2 (0.050)	5 (ND)
Sediment	8.39	1.4	6.58	36.2	0.8 (0.010)	1 (ND)
CAFAS	5.21	5.7	7.56	120.6	**2.6 (0.080)**	3 (ND)
CAFAS	8.39	1.4	7.56	**159.4**	**1.6 (0.080)**	3 (ND)

	Leachate		Eluate			
	pH	EC (µS/cm)	Al (mg/l) (%)	Cr (mg/l) (%)	Cu (mg/l) (%)	Fe (mg/l) (%)
Sediment	5.21	5.7	0.01 (< 0.000)	0.01 (0.009)	**0.10 (0.205)**	< 0.00 (< 0.000)
Sediment	8.39	1.4	0.02 (< 0.000)	0.02 (0.018)	0.06 (0.123)	< 0.00 (< 0.000)
CAFAS	5.21	5.7	0.05 (< 0.000)	**0.01 (0.025)**	0.04 (0.008)	**0.01 (< 0.000)**
CAFAS	8.39	1.4	0.10 (< 0.000)	0.00 (< 0.000)	0.06 (0.013)	< 0.00 (< 0.000)

	Leachate		Eluate			
	pH	EC (µS/cm)	Mn (mg/l) (%)	Ni (mg/l) (%)	Zn (mg/l) (%)	
Sediment	5.21	5.7	0.001 (< 0.000)	0.05 (0.038)	< 0.00 (< 0.000)	
Sediment	8.39	1.4	0.002 (< 0.000)	0.00 (< 0.000)	< 0.00 (< 0.000)	
CAFAS	5.21	5.7	0.001 (< 0.000)	**0.05 (0.154)**	< 0.00 (< 0.000)	
CAFAS	8.39	1.4	0.001 (< 0.000)	< 0.00 (< 0.000)	< 0.00 (< 0.000)	

In bold – the highest value; *ND* no data.

The quantity of compounds leached over the decades, and the cumulative impact, cannot be assessed properly because no unaltered sample of the original material is available for comparison. It is possible that CAFAS had strong negative impacts on biota and groundwater prior to its transformation to its current state, but this has not been documented due to the lack of monitoring in the past. It is worth noting that the mobility of compounds in combustion waste is strongly dependent upon coal properties, combustion technology and the behaviour of trace elements during combustion^2^. The amount of the mobilised fraction previously leached-out in the cave environment thus remains unquantifiable. As long as CAFAS is present on site, however, leaching will continue, especially of compounds whose concentrations remain high and which have demonstrably been mobilised, e.g. copper compounds (Table 3).

### Microbial activity and toxicity

CAFAS and cave sediment contained comparable concentrations of total and cultivable microbial biomass on tested microbiological media (Table 4), but the CAFAS contains higher levels of macronutrients, especially C, but also N and P (Table 1). This can be attributed to the gradual enrichment of the CAFAS with organic debris, infiltrates, and cave sediments, making it an enriched substrate for microbial metabolism. Whereas the cave sediments displayed zero ETSA (electron transport system activity), the CAFAS activity was measured as 0.22 μl O_2_/gDW h. This is close to the lowest value recorded for sediments from the comparable hyporheic zone, ranging from 0.3 to 28.9 μl O_2_/gDW h, based on an allochthonous source of organic matter^30^. These results are consistent with those of other studies, indicating that the organic matter content of sediments is one of the main drivers of microbial metabolic activity^30–33^. Interestingly, in a previous study^34^ the highest toxic effect of Cu in sediments upon microbial activity was expressed at far lower concentrations than those of Cd, Pb and Zn. The Cu eliminated ETSA completely, whereas the other three metals reduced activity by 40–45%. In contrast, the toxic effects of metals such as Cu can be reduced by high concentrations of nutrients or cells, and those of Cd are reduced by clay minerals^35^. Specific properties of cave sediment and CAFAS have different effects on the metabolic activity of microbial populations. CAFAS can be considered active metabolically and, due to its quantity, it is an important part of the cave ecosystem. CAFAS with its transformation became an important microbial habitat in Divaška jama over time due to the physical cave environment, e.g. the presence of dripping water, autochthonous cave sediments, organic debris and infiltrates at high humidity, and the associated microbial activity.

**Table 4:** Microbial biomass and electron transport system activity (ETSA).

Biomass estimator	Sediment	CAFAS
	RLU	
ATP*	2 (0)	8 (2)
Medium	× 10^5^ cells/g DW	
NA	121.0 (14.0)	86.6 (79.4)
R_2_A	93.1 (31.7)	141.7 (60.3)
TA	32.7 (12.3)	18.5 (17.3)
MEA	0.0 (0.0)	2.1 (0.9)
	(µl O_2_/g DW h)	
ETSA	0.00 (0.00)	0.22 (0.03)

Data are mean (standard deviation); *measured in 10^−2^ dilution.

In the ecotoxicity assays, both the cave sediment and the CAFAS exhibited toxicity towards the bacterial indicators *A. fischeri* and *E. coli* (Table 5), which are used commonly in similar environmental studies^36,37^. Interestingly, among the *E. coli* strains tested, the laboratory strain DH5α was more resilient than the *E. coli* isolated from nature. Nevertheless, these ecotoxicological assays do not identify the exact inhibition factor(s) and mechanisms of toxicity, which range from destabilisation of macromolecules through the exchange of essential cofactors, to the blocking of essential functional groups and the production of reactive oxygen species (ROS)^38^. Metal-induced toxicity is commonly attributed to oxidative stress^39–45^; in the current study this could be related to elevated concentrations of As, Cr, Cu, Hg, Ni and Pb (Table 1).

**Table 5:** Assessing toxicity of solid samples based on *A. fischeri* and eluates based on *E. coli* indicator systems.

Sediment		Solid sample	Dilution of eluates			
Indicator	Unit		0	2	5	10
*E. coli* DH5α	mm		6.9 (0.5)	0.0 (0.0)	0.0 (0.0)	0.0 (0.0)
*E. coli* EC78	mm		9.5 (1.5)	8.4 (1.0)	7.8 (0.8)	6.9 (0.4)
*A. fischeri*	%	20				
**CAFAS**						
Indicator	Unit		0	2	5	10
*E. coli* DH5α	mm		6.9 (1.1)	6.5 (0.5)	6.3 (0.3)	6.5 (0.6)
*E. coli* EC78	mm		8.6 (1.0)	8.0 (1.0)	7.6 (0.8)	7.5 (0.7)
*A. fischeri*	%	55				

Data for *E. coli* toxicology assay are mean (standard deviation).

Dripping water introduces new biomass and organic matter into caves^46–48^ and as such can influence microbial activity and leaching when hitting cave sediments. For example, whereas the alkaline pH of dripping water reduces the solubility of Fe, Mn and Mg, it increases the solubility of Al^49^, which represents the major metal component in both the CAFAS and the cave sediment samples (Table 1). Microbial processes such as bioleaching, bioweathering, biodeterioration and methylation increase the mobility of metals significantly^50^, and this too could play an important role in the overall mobilization of metals in CAFAS and cave sediment. Under the given conditions, biosorption and bioaccumulation of organic pollutants and toxic metals, especially As^51^, Ni^52^, Hg^53^ and Cr^54^, all of which express slightly elevated concentrations in Divaška jama, are also a possible prerequisite for adverse effects on trophic levels within cave ecosystems. There are no data relating to the effects of CAFAS on the cave fauna that was frequently observed on the CAFAS surface in Divaška jama, or on how it affected the normal functioning of the cave ecosystem. Finally, in the case of Divaška jama, the overall picture must be taken into consideration, because there are still many natural cave sediment deposits. Although CAFAS represents an additional source of organic matter and energy for the underground ecosystem, it does not belong to vulnerable karst caves. CAFAS was "integrated" into the cave over time and enriched the karst underground, which is commonly considered oligotrophic. Its removal cannot remedy or eliminate the cumulative effect of the leached material in deep karst underground, but it will help restore the original natural conditions.

## Conclusions

The study reports the current status of aged coal ash with slag mixed with cave material. CAFAS is an important external contaminant representing enrichment with inorganic and organic compounds and dispersed pollution for the cave ecosystem. The soluble fraction of metals in the CAFAS leachate is comparatively low, as is also the case in natural cave sediments. Whereas the estimated hazard level of CAFAS is low for biota and groundwater, it still exhibits some toxicity towards bacterial indicator systems. The microbiota associated with CAFAS shows higher metabolic activity in contrast to that in cave sediment, and in this respect it became an important part of the cave ecosystem over time. CAFAS should not be placed in the karst, because it affects the underground not only by its toxicity but also possibly by disturbing the natural energy balance, which should be addressed in future studies. Considering the evident susceptibility of CAFAS to alteration, various management alternatives should be examined thoroughly.

## Methods, techniques, studied material and area descriptions

### Site description

Divaška jama (*Divača Cave*, 45° 40′ 30.61′′ N 13° 57′ 02.36′′ E) discovered in 1884, is located on the SE edge of the Kras Plateau (Classical Karst, Slovenia). The cave is developed in Upper Cretaceous micritic and sparitic limestone. It is 670 m long and 90 m deep, with its entrance at 430 m a.s.l. Large oscillations in climatic parameters are expressed mainly at the entrance shaft. Temperature in the inner part of Divaška jama is about 11 °C all year round, and the relative humidity is close to saturation. The explored dry passages are about 200 m above the current underground watercourse of the Reka River, estimated from the nearby cave Kačna jama^55^. This part of the Divača area displays clearly expressed NW–SE Dinaric faults^56^. Divaška jama is an ancient relict cave that has been brought close to the land surface by the effects of karst denudation; it contains some old but well-preserved fluvial sediments^57^. Speleothems deposited on the sediments are of various ages; some exceed 350 ka, whereas the youngest started to grow at about 16 ka, at the same time as the general changes of climate that followed the last glacial maximum^55^.

When Divaška jama was reopened for tourists in 1987, it was decided to remove the CAFAS (approximately 100 m^3^) completely from the cave and to restore the cave’s natural conditions ^16^. Since then, several cleaning campaigns have been organized to achieve this goal (Fig. 2). In 2009 the cave was equipped with an electric lighting system, and it now attracts about 2000 visitors per year (B. Lozej, personal communication, 24 March 2020).

### Sampling

Coal bottom ash and slag particles are generally not spherical and have sizes from 2 µm to 20 mm. Coal fly ash consists of spherical glassy particles ranging in size from less than 1 µm to more than 1 mm^1^. The CAFAS layers varied from one to ten centimetres in thickness. CAFAS was placed in the inner part of the cave where stable cave conditions prevail. The exact time period when CAFAS was placed in the particular part of the cave and its age could not be determined. A mixed sample of CAFAS (~ 500 g) containing the upper compressed layer (~ 2 cm), the middle layer (~ 5 cm) and the lower layer (~ 2 cm) at the contact with the limestone bedrock from the tourist trail was collected aseptically during a cleaning campaign. In addition, a sample of naturally deposited fluvial cave sediment (~ 500 g) was collected from a profile deposited in the cave during a timeframe from pre-0.97 Ma to 0.73 Ma^58^.

Sampling of the cave material was approved by the Slovenian Environment Agency, Ministry of the Environment and Spatial Planning, Republic of Slovenia (No. 35602-3/2020-4).

### Analyses of cave samples

The dry weight (DW) in the samples was determined gravimetrically after 5 h drying at 105 °C. The pH value was measured with 0.01 M CaCl_2_. C (organic combustible substances), N, P, S, Na, Ca, Mg, K, As, Al, Be, Cd, Co, Cr, Cu, Fe, Hg, Mn, Ni, Pb, V and Zn, as well as the ash content, were analysed at AGRO-LA, Jindřichův Hradec (Czech Republic) according to the standard methods of the Czech Central Institute for Supervising and Testing in Agriculture^59^. The amount of particulate organic matter (POM) was determined as loss on ignition at 530 °C for 3 h.

Radiation levels of the CAFAS and the cave sediments were measured in a laboratory, using a Radiation Alert Ranger (SE International) and expressed in cpm (counts per minute). The unit measures alpha, beta, gamma and X-ray radiation (ionizing radiation only). Specific activity of radionuclides was assessed using high-resolution gamma spectrometry (DP-LMSAR-09) and performed at the Institute of Occupational Safety, Ljubljana (Slovenia).

X-ray powder diffraction (XRD) analysis of materials was performed at the National Institute of Chemistry, Ljubljana (Slovenia) using a PANalytical X’Pert PRO diffractometer with CuKα_1_ radiation (1.54060 Å). Samples were loaded into a flat disc-like sample holder. The XRPD data were collected in the 2Θ range from 5 to 80° 2Θ, and the qualitative analysis of collected XRD patterns was performed using the X’Pert HighScore Plus Suite^60^.

### Analyses of eluates

Samples of CAFAS and cave sediment were wrapped in cellulose wadding in perforated polyester bags, which were attached inside beakers of water that would leach out any soluble compounds. Waters with pH values of 8.39 and 5.21 (10% HCl was used for pH adjustment) were used as leachate. A magnetic stirrer (400 RPM) was used to create a vortex within the leaching solution (10% w/v) during overnight extraction. These conditions were selected to maximize the extraction efficiency.

After passing the eluate through a filter (MN-617, Macherey–Nagel) and centrifuging at 4000 RPM for 10 min, some coarse particles remained. Additional steps of centrifuging at 14,000 RPM for 3 min and filtration using a 0.45 µm pore-size filter (Chromafil® Xtra H-PTFE-45/25, Macherey–Nagel) were required to obtain a clear solution for chemical analysis, using a YSI 9300 spectrophotometer (YSI), for determination of Al, Cl, Cr, Cu, Fe, K, Mn, Ni and Zn. The eluate was analysed also for pH and electrical conductivity – EC (Multi 3620 IDS, WTW).

### Ecotoxicity assessment

The toxicity of the solid samples was assessed using an S3 Environmental Toxicity testTM kit (S3.1 Environmental Toxicity Test, Wilton Biotechnologies, UK). The methodology is based upon the inhibition of light emission by the bioluminescent bacterium *Aliivibrio fischeri* after contact with a toxic substance.

In addition, the toxicity was investigated using a disc diffusion agar method and the bacterium *Escherichia coli* as indicator organism. Two strains were used, DH5α [*endA1 hsdR17 supE44 thi-1 recA1 gyrA96 relA1 Δlac(lacZYA-argF) (φ80 lacZ ΔM15)*], a strain frequently used in molecular biology^61,62^, and EC78, a strain isolated from a cave. Any microorganisms remaining in the solution were removed by sterile filtration through a 0.22 μm pore-size filter (Durapore®, Merck Millipore). Sterile filter discs with a diameter of 6 mm (Filtrak 388, Spezialpapierfabrik Niederschlag, Germany) were dipped in concentrated (10% w/v) and diluted samples (2×, 5×, 10×, prepared in sterile deionised water) and placed on LB agar plates (Luria broth base, Sigma), which were pre-inoculated with a fresh overnight culture of *E. coli*. Growth inhibition zones were measured after 48 h incubation at 37 °C.

### Microbial biomass and activity

The total microbial biomass was estimated as total adenosine triphosphate (ATP) (AquaSnapTotal, Hygiena, USA) in 10^–2^ dilutions and expressed in RLU (Relative-Light Units).

To estimate the concentration of cultivatable microorganisms, cave samples were homogenised in 0.9% physiological saline solution, serially diluted (up to 10^–3^) and plated onto nutrient agar (NA, Sigma-Aldrich); R2A agar (Merck); malt extract agar (MEA, Sigma-Aldrich); tap water agar (TWA) containing 1.5% agar (Biomérieux). Petri dishes were cultivated aerobically at 20 °C for 14 days. Viable cell counts were expressed as colony forming units (CFU) per DW.

Respiratory electron transport system activity (ETSA) was determined based upon reaction between NADPH/NADH and 2-(p-iodophenyl)-3-(p-nitrophenyl)-5-phenyl tetrazolium chloride at 20 °C for 60 min, resulting in formation of an iodonitrotetrazolium (formazan). Formazan production was measured by applying the assay originally proposed by Packard (1971)^63^ and modified by G.-Toth (1999)^64^. Formazan was determined spectrophotometrically at 490 nm and converted to oxygen used per dry weight in a given time interval, i.e. µl O_2_/g DW h^65^.

## References

[1] Siddique R (2010). Utilization of coal combustion by-products in sustainable construction materials. Resour. Conserv. Recycl..

[2] Querol X, Juan R, Lopez-Soler A, Fernandez-Turiel J, Ruiz C (1996). Mobility of trace elements from coal and combustion wastes. Fuel.

[3] Kalkreuth W (2014). Evaluation of environmental impacts of the Figueira coal-fired power plant, Paraná, Brazil. Energ. Explor. Exploit..

[4] MacBride S (2013). The archeology of coal ash: An industrial-urban solid waste at the dawn of the hydrocarbon economy. IA. Soc. Ind. Archeol..

[5] Matzenbacher C (2017). DNA damage induced by coal dust, fly and bottom ash from coal combustion evaluated using the micronucleus test and comet assay in vitro. J. Hazard. Mater..

[6] Messinger M, Silman M (2016). Unmanned aerial vehicles for the assessment and monitoring of environmental contamination: An example from coal ash spills. Environ. Pollut..

[7] Santamarina J, Torres-Cruz L, Bachus R (2019). Why coal ash and tailings dam disasters occur. Science.

[8] Ruhl L (2009). Survey of the potential environmental and health impacts in the immediate aftermath of the coal ash spill in Kingston, Tennessee. Environ. Sci. Technol..

[9] Vengosh A (2019). Evidence for unmonitored coal ash spills in Sutton Lake, North Carolina: Implications for contamination of lake ecosystems. Sci. Total Environ..

[10] Ramsey A, Faiia A, Szynkiewicz A (2019). Eight years after the coal ash spill: Fate of trace metals in the contaminated river sediments near Kingston, eastern Tennessee. Appl. Geochem..

[11] Schwartz G (2016). Leaching potential and redox transformations of arsenic and selenium in sediment microcosms with fly ash. Appl. Geochem..

[12] Giam X, Olden J, Simberloff D (2018). Impact of coal mining on stream biodiversity in the US and its regulatory implications. Nat. Sustain..

[13] Kravchenko J, Lyerly H (2018). The impact of coal-powered electrical plants and coal ash impoundments on the health of residential communities. N. C. Med. J..

[14] Greeley M, Adams S, Elmore L, McCracken M (2016). Influence of metal(loid) bioaccumulation and maternal transfer on embryo-larval development in fish exposed to a major coal ash spill. Aquat. Toxicol..

[15] Lemly A (2018). Environmental hazard assessment of coal ash disposal at the proposed Rampal power plant. Hum. Ecol. Risk Assess..

[16] Puc D (1999). Divaška Jama.

[17] Batty L (2005). The potential importance of mine sites for biodiversity. Mine Water Environ..

[18] Luo Z (2020). Adaptive development of soil bacterial communities to ecological processes caused by mining activities in the Loess Plateau, China. Microorganisms.

[19] Thavamani P (2017). Microbes from mined sites: Harnessing their potential for reclamation of derelict mine sites. Environ. Pollut..

[20] Apostolova D, Bechtel A, Kostova I, Stefanova M (2020). Biomarkers assemblage of unburned coal particles in fly ashes from Bulgarian thermoelectric power plants. J. Min. Geol. Sci..

[21] Cui Y (2021). Stoichiometric models of microbial metabolic limitation in soil systems. Global Ecol. Biogeogr..

[22] Dean W (1974). Determination of carbonate and organic matter in calcareous sediments and sedimentary rocks by loss on ignition: comparison with other methods. J. Sediment. Petrol..

[23] MacDonald D, Ingersoll C, Berger T (2000). Development and evaluation of consensus-based sediment quality guidelines for freshwater ecosystems. Arch. Environ. Contam. Toxicol..

[24] Milačič R, Zuliani T, Vidmar J, Oprčkal P, Ščančar J (2017). Potentially toxic elements in water and sediments of the Sava River under extreme flow events. Sci. Total Environ..

[25] Vidmar J (2017). Elements in water, suspended particulate matter and sediments of the Sava River. J. Soils Sediments.

[26] Oprčkal P (2020). Remediation of contaminated soil by red mud and paper ash. J. Clean. Prod..

[27] Nisnevich M, Sirotin G, Schlesinger T, Eshel Y (2008). Radiological safety aspects of utilizing coal ashes for production of lightweight concrete. Fuel.

[28] Komonweeraket K, Cetin B, Benson C, Aydilek A, Edil T (2015). Leaching characteristics of toxic constituents from coal fly ash mixed soils under the influence of pH. Waste Manage..

[29] Popovic A, Djordjevic D (2009). pH-dependent leaching of dump coal ash: Retrospective environmental analysis. Energ. Source Part A.

[30] Mori N, Simčič T, Žibrat U, Brancelj A (2012). The role of river flow dynamics and food availability in structuring hyporheic microcrustacean assemblages: A reach scale study. Fundam. Appl. Limnol..

[31] de Vicente I, Amores V, Guerrero F, Cruz-Pizarro L (2010). Contrasting factors controlling microbial respiratory activity in the sediment of two adjacent Mediterranean wetlands. Naturwissenschaften.

[32] Mori N, Simčič T, Brancelj A, Robinson C, Doering M (2017). Spatiotemporal heterogeneity of actual and potential respiration in two contrasting floodplains. Hydrol. Process..

[33] Muri G, Simčič T (2004). Respiratory activity in sediments of three mountain lakes in the Julian Alps and in subalpine Lake Bled (Slovenia): Effects of altitude and anthropic influence. Aquat. Microb. Ecol..

[34] Broberg A (1983). Effects of heavy metals on electron transport system activity (ETSA) in freshwater sediments. Ecol. Bull..

[35] Aljerf L, AlMasri N (2018). A gateway to metal resistance: Bacterial response to heavy metal toxicity in the biological environment. AAC.

[36] Robbens J, Dardenne F, Devriese L, De Coen W, Blust R (2010). *Escherichia coli* as a bioreporter in ecotoxicology. Appl. Microbiol. Biotechnol..

[37] Abbas M (2018). *Vibrio fischeri* bioluminescence inhibition assay for ecotoxicity assessment: A review. Sci. Total Environ..

[38] Prabhakaran P, Ashraf M, Aqma W (2016). Microbial stress response to heavy metals in the environment. RSC Adv..

[39] Hughes M (2002). Arsenic toxicity and potential mechanisms of action. Toxicol. Lett..

[40] Cervantes C (2001). Interactions of chromium with microorganisms and plants. FEMS Microbiol. Rev..

[41] Ladomersky E, Petris M (2015). Copper tolerance and virulence in bacteria. Metallomics.

[42] Miller D, Woods J (1993). Redox activities of mercury-thiol complexes: Implications for mercury-induced porphyria and toxicity. Chem Biol. Interact..

[43] Macomber L, Hausinger R (2011). Mechanisms of nickel toxicity in microorganisms. Metallomics.

[44] Lopes, A., Peixe, T., Mesas, A. & Paoliello, M. in *Reviews of Environmental Contamination and Toxicology.* **236** 193–238 (Springer, 2016). 10.1007/978-3-319-20013-2_3 26423075

[45] Valko M, Morris H, Cronin M (2005). Metals, toxicity and oxidative stress. Curr. Med. Chem..

[46] Simon K, Pipan T, Culver D (2007). A conceptual model of the flow and distribution of organic carbon in caves. J. Cave Karst Stud..

[47] Mulec J, Oarga-Mulec A (2016). ATP luminescence assay as a bioburden estimator of biomass accumulation in caves. Int. J. Speleol..

[48] Gerič B, Pipan T, Mulec J (2004). Diversity of culturable bacteria and meiofauna in the epikarst of Škocjanske jame Caves (Slovenia). Acta Carsol..

[49] Adibee N, Osanloo M, Rahmanpour M (2013). Adverse effects of coal mine waste dumps on the environment and their management. Environ. Earth Sci..

[50] Jing R, Kjellerup B (2018). Biogeochemical cycling of metals impacting by microbial mobilization and immobilization. J. Environ. Sci..

[51] Huang J (2016). Arsenic trophodynamics along the food chains/webs of different ecosystems: A review. Chem. Ecol..

[52] Cempel, M. & Nikel, G. Nickel: A review of its sources and environmental toxicology. *Pol. J. Environ. Stud.* **15** (2006).

[53] Rimmer C, Miller E, McFarland K, Taylor R, Faccio S (2010). Mercury bioaccumulation and trophic transfer in the terrestrial food web of a montane forest. Ecotoxicology.

[54] Fernández P, Viñarta S, Bernal A, Cruz E, Figueroa L (2018). Bioremediation strategies for chromium removal: Current research, scale-up approach and future perspectives. Chemosphere.

[55] Mihevc, A. *Speleogeneza Divaškega Krasa*. Vol. 27 180 (Založba ZRC, 2001).

[56] Žvab Rožič, P., Čar, J. & Rožič, B. Geological structure of the Divača area and its influence on speleogenesis and hydrogeology of Kačna jama. *Acta Carsol.* **44** (2015).

[57] Zupan Hajna, N., Mihevc, A., Pruner, P. & Bosák, P. *Palaeomagnetism and Magnetostratigraphy of Karst Sediments in Slovenia*. Vol. 8 (Založba ZRC, ZRC SAZU, 2008).

[58] Bosák P, Pruner P, Zupan Hajna N (1998). Palaeomagnetic research of cave sediments in SW Slovenia. Acta Carsol..

[59] Zbíral, J. *Soil Analyses, Part 1 (in Czech)*. (Central Institute for Supervising and Testing in Agriculture, 1995).

[60] Degen T, Sadki M, Bron E, Konig U, Nenert G (2014). The HighScore suite. Powder Diffr..

[61] Chan W, Verma C, Lane D, Gan S (2013). A comparison and optimization of methods and factors affecting the transformation of *Escherichia coli*. Biosci. Rep..

[62] Kostylev, M., Otwell, A., Richardson, R. & Suzuki, Y. Cloning should be simple: *Escherichia coli* DH5α-mediated assembly of multiple DNA fragments with short end homologies. *PLoS ONE* **10** (2015). 10.1371/journal.pone.0137466 PMC4562628 26348330

[63] Packard T (1971). The measurement of respiratory electron transport activity in marine phytoplankton. J. Mar. Res..

[64] G.-Toth, L. in *Biologische Gewässeruntersuchung. Methoden der Biologische Wasseruntersuchung 2.* (eds W von Tumpling & G Friedrich) 465–473 (Gustav Fischer Verlag, 1999).

[65] Kenner R, Ahmed S (1975). Measurements of electron transport activities in marine phytoplankton. Mar. Biol..

